# Lupin, a Unique Legume That Is Nodulated by Multiple Microsymbionts: The Role of Horizontal Gene Transfer

**DOI:** 10.3390/ijms24076496

**Published:** 2023-03-30

**Authors:** Abdelhakim Msaddak, Mohamed Mars, Miguel A. Quiñones, M. Mercedes Lucas, José J. Pueyo

**Affiliations:** 1 Department of Soil. Plant and Environmental Quality, Institute of Agricultural Sciences, ICA-CSIC, 28006 Madrid, Spain; 2 Laboratory of Biodiversity and Valorization of Arid Areas Bioresources, BVBAA, Faculty of Sciences, University of Gabès, Erriadh, Zrig, Gabès 6072, Tunisia

**Keywords:** *Lupinus*, Bradyrhizobium, rhizobia, 16S rRNA, *nodC*, nifH, symbiotic genes, horizontal gene transfer

## Abstract

Lupin is a high-protein legume crop that grows in a wide range of edaphoclimatic conditions where other crops are not viable. Its unique seed nutrient profile can promote health benefits, and it has been proposed as a phytoremediation plant. Most rhizobia nodulating *Lupinus* species belong to the genus *Bradyrhizobium,* comprising strains that are phylogenetically related to *B. cytisi, B. hipponenese, B. rifense, B. iriomotense/B. stylosanthis, B. diazoefficiens, B. japonicum, B. canariense/B. lupini*, and *B. retamae/B. valentinum*. Lupins are also nodulated by fast-growing bacteria within the genera *Microvirga*, *Ochrobactrum*, *Devosia*, *Phyllobacterium, Agrobacterium, Rhizobium*, and *Neorhizobium*. Phylogenetic analyses of the *nod* and *nif* genes, involved in microbial colonization and symbiotic nitrogen fixation, respectively, suggest that fast-growing lupin-nodulating bacteria have acquired their symbiotic genes from rhizobial genera other than *Bradyrhizobium*. Horizontal transfer represents a key mechanism allowing lupin to form symbioses with bacteria that were previously considered as non-symbiotic or unable to nodulate lupin, which might favor lupin’s adaptation to specific habitats. The characterization of yet-unstudied *Lupinus* species, including microsymbiont whole genome analyses, will most likely expand and modify the current lupin microsymbiont taxonomy, and provide additional knowledge that might help to further increase lupin’s adaptability to marginal soils and climates.

## 1. Introduction

*Fabaceae* or *Leguminoseae* (*nom. cons.*) constitute a large plant family, widespread all over the world, that includes more than 19,700 species [1,2]. Many legume species, including all the agronomically important legume crops, establish symbiotic associations with soil bacteria that are able to fix atmospheric nitrogen (N_2_) in specific symbiotic organs called nodules. The legume-nodulating bacteria, known as rhizobia, are gram-negative bacteria that have the ability to induce the formation of a de novo organ in the roots and/or stems of leguminous plants [3]. They are capable of fixing atmospheric N_2_ owing to the microaerobic environment that is created inside the nodule. Most rhizobia belong to the alpha subgroup of the phylum *Pseudomonadota* (formerly *Proteobacteria*). The class *α-Proteobacteria* comprises most of the N_2_-fixing genera that nodulate legumes, while some symbiotic genera belong to the class *β-Proteobacteria*. The great diversity of legume species is reflected in their wide variety of nodulating microsymbionts.

Ribosomal ribonucleic acid (rRNA) is the primary component of ribosomes, and is essential to all cells. rRNA is a ribozyme that facilitates protein synthesis in the ribosomes [4]. The 16S rRNA gene is present in all bacteria, and phylogenetic analyses based on these gene sequences are used to determine genetic relationships among organisms. Including conserved and variable regions, the 16S rRNA gene has a considerable size (around 1500 bp), which provides sufficient information for taxonomy. This gene presents a high level of conservation at the species level in such a way that the sequence variations observed in this gene can provide a precise indicator of evolution. For these characteristics, the 16S rRNA gene is considered as a primary discriminative taxonomic marker to classify a strain at the genus and often at the species level, and represents one of the preferred genetic methods for bacterial phylogenetic analysis [5,6,7], including the phylogenetic classification of legume microsymbionts [8,9,10]. 

Simultaneously with the development of molecular biology techniques, bioinformatics tools, and whole genome sequencing, a large number of 16S rRNA gene sequences are available in gene databases. The DNA DataBank of Japan (DDBJ), the European Nucleotide Archive (ENA), and the National Center for Biotechnology Information (NCBI) gene databases, which are part of the International Nucleotide Sequence Database Collaboration (INSDC), constitute a potent tool for scientists, as 16S rRNA gene sequences are available for the construction of phylogenetic trees and identification of species [11]. Phylogenetic trees are useful for structuring taxonomic classifications, and they provide information about events that might have occurred during evolution, showing lines of evolutionary descent for diverse species, organisms, or even genes. Phylogenetic tree analyses affiliate new bacterial isolates with their closest described strains [9,10,12]. Despite the fact that the aforementioned databases exchange data on a daily basis, multiple sequence uploads have often led to lack of agreement on the dependability of 16S rRNA gene sequence data [13,14]. 

Phylogenetic analysis of the nearly full-length sequences of the 16S rRNA genes of the majority of representative microsymbiont strains has allowed for the identification of 18 genera and around 238 species of bacteria that nodulate legumes [15]. Within the class *α-Proteobacteria*, the family *Rhizobiaceae* comprises the genera *Rhizobium*, *Pararhizobium, Allorhizobium*, *Ensifer* (syn. *Sinorhizobium*), *Neorhizobium,* and *Shinella*. The genera *Aminobacter, Mesorhizobium,* and *Phyllobacterium* are included in the family *Phyllobacteriaceae*. The genera *Microvirga* and *Methylobacterium* are part of the family *Methylobacteriaceae*. The genera *Bradyrhizobium*, *Blastobacter,* and *Photorhizobium* constitute the family *Bradyrhizobiaceae*. The genus *Ochrobactrum* is specific to the *Brucellaceae.* In the family *Hyphomicrobiaceae*, only *Devosia* has been reported to nodulate Fabaceae [12]. In the class *β-Proteobacteria*, the genus *Cupriavidus,* along with *Burkholderia,* are part of the family *Burkholderiaceae* [16]. Bacteria of the *γ-Proteobacteria* group have also been detected in legume nodules [17,18]; however, later reports have questioned their ability to nodulate legumes [19]. 

Effective nodulation and nitrogen fixation are supported by the *nod* and *nif* gene clusters, respectively. Nitrogen is one of the most important nutrients for plant growth. Nodule organogenesis is induced by the rhizobial-secreted Nod factors, which are signal molecules controlled by the *nod* genes that are recognized by the host plant and lead to the formation of the symbiotic nodule [20]. This suggests that *nod* genes are responsible for determining the specificity of the legume–rhizobial symbiosis [21]. The *nif* genes, which codify for the nitrogenase enzyme complex subunits and several regulatory proteins, are essential for nitrogen fixation [22]. In consequence, while 16S rRNA gene sequences are key for the classification of bacterial species and a reliable marker for phylogenetic analyses, a multigene analysis combining the 16S rRNA and *nod* and *nif* genes [23] tends to be used to approximate the systematic characteristics of rhizobia. 

The nif and nod genes are often carried on symbiotic islands in the chromosome or in plasmids, which are easily transferred horizontally between different bacterial species within and across genera [24,25]. This transfer of *nod* and *nif* genes between bacteria in the rhizosphere can confer the ability to nodulate legumes to non-rhizobial species [26,27]. It has been suggested that stressful environments, in general, may favor horizontal gene transfer, as the acquisition of certain genes might represent an adaptive advantage. Furthermore, bacteria can engage in horizontal gene transfer to rapidly disseminate traits in a population, thus providing indiscriminate benefits to their neighbors, by means of a behavior that can be considered a method of bacterial cooperation [28]. In this manner, the transfer of symbiotic genes to bacteria that are well-adapted to local soil conditions allows these microorganisms to become rhizobial symbionts of originally incompatible legumes [29]. The horizontal transfer of symbiotic genes most often leads to phylogenies that differ from those of the core genome of the receiving bacteria [21,24].

There is abundant evidence that horizontal gene transfer occurs commonly in nature, involving all kinds of transposable elements and all classes of living organisms [30,31]. Even phylogenetically distant species may experience the transference of transposable elements to/from their genomes through transfer vectors such as viruses, bacteria, or other parasites [32]. There are many examples in the literature of how horizontal gene transfer has driven significant evolutionary changes, or helped organisms to adapt to abiotic stress [33]. There are also some examples of horizontal gene transfer helping plants to adapt to biotic stress: for example, when symbiotic genes are acquired by bacteria with PGPR traits [34]. It is well known that biofilms facilitate horizontal gene transfer between bacteria and that soil bacteria are often organized in biofilms on root, litter, and soil particles [35]. Therefore, biofilms may play an important role in the movement of transposable elements between soil organisms.

Initially identified within a species, biological variants, commonly named biovars, have been described in diverse bacterial species. A biovar represents a specific group of bacterial strains which are distinguishable from other strains in the same species on the basis of biochemical or physiological characteristics. Biovars were described for the first time in *Rhizobium leguminosarum* by Jordan [36] in a taxonomical revision of rhizobial species. In rhizobia, biovars and symbiotic variants, or symbiovars, are used to differentiate distinct subgroups, usually within a genus, comprising species that nodulate the same legume [37]. Legume host range specificity is encoded in rhizobial genetic elements that can be disseminated by horizontal transfer; thus, symbiovars can be shared by rhizobial species belonging to different genera [9,37]. 

Until the present century, lupin was assumed to be a microsymbiont-specific legume. However, owing to the evolution of molecular taxonomy, nowadays, lupin can be considered as a promiscuous legume that can be nodulated by different *Bradyrhizobium* lineages [38,39,40], and by bacteria within the genera *Phyllobacterium* [41,42], *Ochrobactrum* [27], *Rhizobium, Neorhizobium, Agrobacterium* [43], *Microvirga* [42,44], and *Devosia* [12]. The aim of this review was to collect the existing information on the diversity and taxonomy of the rhizobial bacteria associated with *Lupinus* spp. nodules. Phylogenetic trees, including the published 16S rRNA, *nodC,* and *nifH* gene sequences of lupin microsymbionts, will be analyzed. The incongruences between the classifications derived from the 16S rRNA gene and the symbiotic gene sequences analyses, and the symbiovars that can be defined thereafter, will be discussed. The importance of lupin, a unique legume with specific characteristics, as summarized below, substantiates the significance of the present review. Moreover, the fact that different lupin-nodulating bacteria might be able to modulate or even determine some legume agronomic traits, such as its adaptability to different soils, its tolerance to abiotic stress, or the nutrient profile of its grain adds relevance to the current knowledge on lupin microsymbionts, and to the discussion on the role that horizontal gene transfer appears to play. 

## 2. Lupin, a Unique Legume in So Many Ways

The legume genus *Lupinus* (*Papilionideae*: *Genisteae*) comprises 200–500 species of perennial and annual shrubs and herbs with an amphi-Atlantic distribution [1]. *Lupinus* species are distributed in Europe; the Near East; North and East Africa; and North, Central, and South America. Less than 13 species are native of the Old World, mainly surrounding the Mediterranean basin, while American species are much more numerous [45,46]. Lupins have been grown since antiquity and represent an important pulse crop due to the highly effective N_2_-fixing symbiosis they establish with rhizobia, as well as their adaptation to different climates and soils. 

Lupins are mainly employed as a source of protein in both human and animal nutrition. The high protein content of lupin seeds (up to 44% dry weight) is similar to that of soybean, and lupin has been proposed as a viable alternative to soybean cultivation in Europe [47]. Lupin seeds possess a high nutritional value and unique phytochemical profile and biological activities [48,49]. Among the lupin species, *L. albus* (white lupin), *L. luteus* (yellow lupin), *L. angustifolius* (blue lupin), and *L. mutabilis* (tarwi) have gained agricultural market importance and have become part of the modern agriculture and food systems for high-protein production [47]. *Lupinus* species have also been studied as medicinal plants [50] and used in traditional medicine to treat various diseases, including urinary tract infections, heart conditions, or skin disorders [51]. Due to the presence of essential fatty acids, specific proteins, and amino acids, as well as minerals, alkaloids, and dietary fiber in lupin seeds, component fractionation allows for extracts to be obtained that display promising anticarcinogenic, antidiabetic, antihypertensive, anti-inflammatory, antimicrobial, and antioxidant activities [51]. The phenolic compounds and flavonoid constituents in lupin seed extracts have important antioxidant activity [51,52]. Lupin protein hydrolysates present anti-inflammatory properties [53]. The antifungal and antibacterial activities have been demonstrated in different studies that report an inhibitory effect toward various bacterial and fungal pathogens [54,55].

Lupin plants have also been shown to have great potential for the recovery of degraded soils and in heavy metal phytoremediation [56,57,58,59]. *Lupinus albus* appears to be an especially well-suited species to withstanding heavy metal stress, and white lupin plants inoculated with a mercury-tolerant *Bradyrhizobium* strain have been reported to present increased mercury tolerance [59]. Additionally, its adaptive mechanisms make white lupin a choice crop for acidic soils affected by aluminum toxicity [60]. Nitrogen and phosphorus are, together with potassium, the two principal nutrients needed by plants, and are essential to ensure crop yield. In phosphate-deficient soils, some lupin species, including white lupin, are able to modify their root architecture to form cluster roots, which are bottlebrush-like structures consisting of hundreds of short rootlets [61]. Cluster roots display enhanced synthesis and secretion of acid phosphatases, organic acids, flavonoids, and proton efflux. Such modifications lead to the mobilization of the soil’s unavailable phosphorus. Therefore, lupin arises as a choice crop for nutrient-poor soils, as it reduces the need for both N and P fertilizers [61]. Lupin’s ability to adapt to acid, nutrient-poor, or moderately polluted soils, and to arid and cold climates, enables lupin cultivation in affected areas, where other more demanding crops, such as soybeans, are not economically viable [62]. Identifying lupin cultivars tolerant to a variety of abiotic stresses may allow for the expansion of lupin cultivation to a broader range of edaphoclimatic conditions [47]. 

Lupin nodules are also unique. According to the host legume and independently of the microsymbiont species, nodules can be determinate or indeterminate [21,63]. Determinate nodules present a transient meristem, while indeterminate nodules maintain meristematic activity throughout their lifespan. All genera examined in the *Fabaceae* subfamilies *Mimosoideae* and *Caesalpinioideae,* and most tribes within the subfamily *Papilionoideae,* form indeterminate nodules, while the papilionoid species within the tribes *Desmodieae*, *Dalbergieae*, *Psoraleeae*, *Phaseoleae*, and some genera in the *Loteae* form determinate nodules [63]. The lupinoid nodule is a subtype of indeterminate nodule, first identified in lupin [64], and to our knowledge has only been described in another legume genus, *Listia* (*Papilionideae*: *Crotalarieae*) [65]. The lupinoid nodule is characterized by the presence of lateral meristems that allow the nodule to encircle the root. Another characteristic of the lupinoid nodule is that the infected cells remain mitotically active and the symbiosomes, much like other cell organelles, are equally distributed between the daughter cells [66,67]. The infection process that leads to lupin nodule organogenesis is also uncommon, as rhizobia infection does not occur through an infection thread, as happens in most legumes. Rhizobia enter the root intercellularly and specifically infect a cell beneath a root hair [66]. Remarkably, in the work in which the infection process was first described, and despite the fact that lupin was thought to be a microsymbiont selective legume exclusively nodulated by *Bradyrhizobium* sp. (*Lupinus*), a *Mesorhizobium loti* strain was used that was able to elicit nodules, but unable to form effective nitrogen-fixing nodules [66].

## 3. Rhizobia That Nodulate Lupins, Many More Than Initially Expected

**Figure 1:**
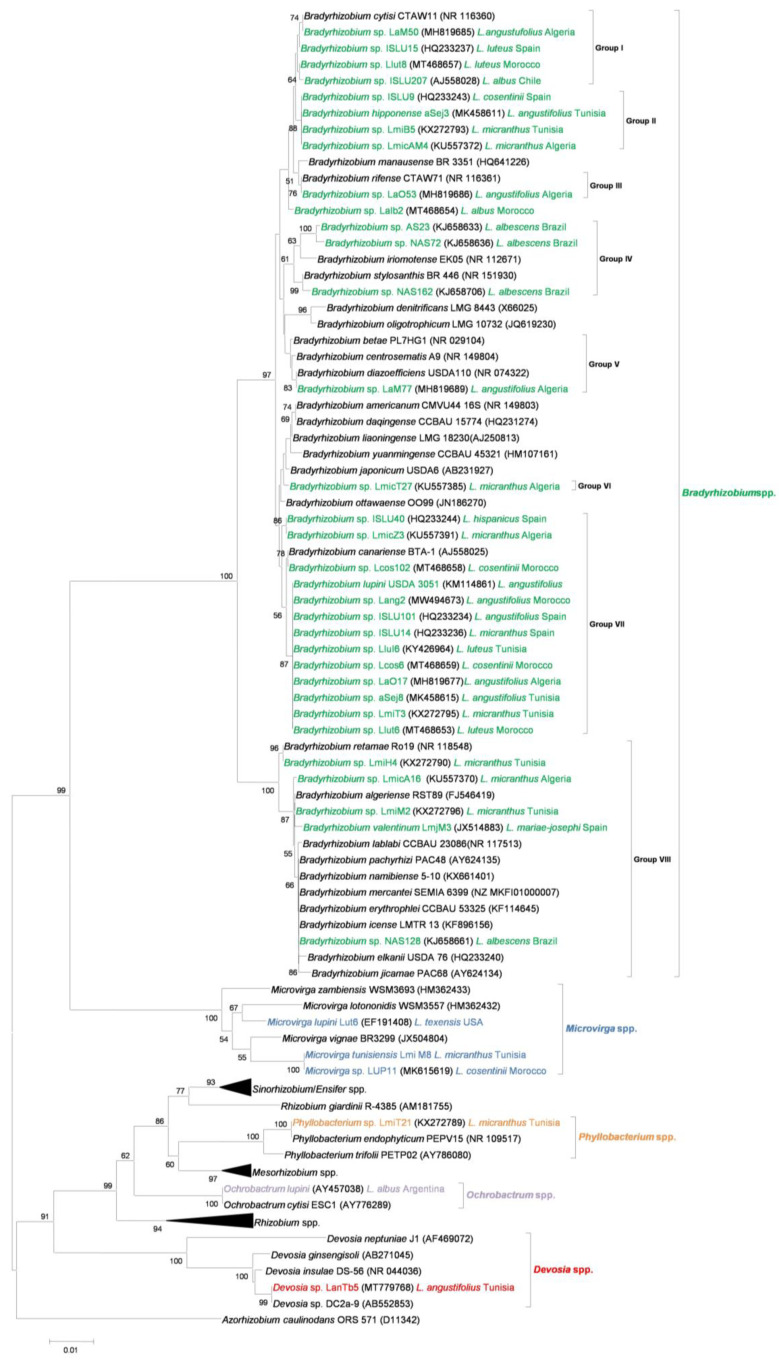
Neighbor-joining phylogenetic analysis of 16S rRNA gene sequence of different species of *Lupinus* spp. microsymbionts and their closest relatives. Lupin microsymbionts are highlighted in color as follows: *Bradyrhizobium* spp. in green; *Microvirga* spp. in blue; *Phyllobacterium* spp. in orange; *Ochrobactrum* spp. in yellow; *Devosia* spp. in red. Values of bootstrap probability greater than 50% are indicated. The host legume and place of isolation are reported when known.

Lupin is nowadays described as a promiscuous plant host, as it can be nodulated by many different species and genotypes of symbiotic bacteria [40]. Until recently, lupins were assumed to be exclusively nodulated by slow-growing rhizobia of the genus *Bradyrhizobium*, initially referred to as *Bradyrhizobium* sp. (*Lupinus*) or *B. lupini* [68,69]. Numerous species under the genus *Bradyrhizobium* have thereafter been described as lupin microsymbionts: *B. lupini* [3,42,70,71], *B. canariense, B. japonicum* [38,42,70,72,73,74,75], *B. valentinum* [76], *B. elkanii* [77], *B. cytisi* [70], *B. diazoefficiens* [70], and *B. hipponense* [78]. Figure 1 shows the phylogenetic analysis of the 16S rRNA gene sequence of some examples of different species of *Lupinus* spp. microsymbionts and their closest relatives. Recently, two new putative *Bradyrhizobium* genospecies within the *genistearum* symbiovar were identified [79,80]. As could be expected, all lupin-nodulating species in the genus *Bradyrhizobium* are phylogenetically close, according to a 16S rRNA gene sequence-derived tree (Figure 1).

*Lupinus mariae-josephi,* the last *Lupinus* species discovered in the Old World, is an endemic species, growing in alkaline soils in Eastern Spain [81]. Remarkably, this species, unlike other lupins, thrives in high-pH lime soils [82]. The phylogenetic analysis of 16S rRNA gene sequences of the rhizobia isolated from *L. mariae-josephi* nodules evidenced a new species, *B. valentinum,* belonging to a distinct evolutionary lineage that also includes *B. jicamae* [39,83], and which is phylogenetically distant from any other lineage of lupin microsymbionts isolated in the Iberian Peninsula. Considering that most New World lupin species microsymbionts have not yet been characterized, the number of new *Bradyrhizobium* species able to nodulate lupins might substantially increase in the future. The *Bradyrhizobium* strains isolated in Europe from *Lupinus* spp. also prevail in the soils of South Africa and Western Australia as a result of their introduction with lupin seeds [74]. Housekeeping gene trees reveal a relatively high level of diversity among Old World lupin-nodulating bradyrhizobia, but most isolates are placed in the *B. japonicum* lineage, and some are closely related to *B. canariense* [72,74]. 

Despite the prevalence of slow-growing *Bradyrhizobium* species, fast-growing bacteria belonging to the genera *Phyllobacterium* [41,42,71], *Ochrobactrum* [27], *Microvirga* [42,44,71,84], *Rhizobium*, *Neorhizobium, Agrobacterium* [43], and *Devosia* [12] have also been described as lupin microsymbionts. *Burkholderia*, a genus that nodulates other legume species, has also been isolated from *L. perennis* nodules [85]; however, its symbiotic effectiveness after re-inoculation has yet to be confirmed. Phylogenetic analyses based on the 23S and 16S rRNA gene sequences of fast-growing bacteria isolated from *L. honoratus* nodules affiliated them to the genus *Ochrobactrum,* and classified a new species (*O. lupini*) [27]. They were able to nodulate *L. albus* and fix nitrogen. A *Phyllobacterium* strain, isolated from *Trifolium pretense* and classified as a new species (*P. trifolii*), was reported to nodulate *L. albus* [41]. A *Phyllobacterium* strain was also isolated from *L. micranthus* nodules [42]. Two studies have reported that *Microvirga* sp. strains were able to nodulate *L. micranthus* [42,71] and *L. cosentinii* [44]. The 16S rRNA gene sequence of those isolates showed high nucleotide identity (up to 98%) with other species in the genus *Microvirga*. The isolates were classified as a new species (*M. tunisiensis*) [9]. *Microvirga* bacteria have also been isolated from *L. texensis* nodules and classified as a new species (*M. lupini*) [84] according to a phylogenetic analysis based on 16S rRNA gene sequences. Msaddak et al. [12] reported that *Devosia* sp. was able to nodulate *L. micranthus.* A phylogenetic analysis of the concatenated *dnaK*, *atpD,* and *recA* gene sequences led to the classification of various bacterial strains isolated from *L. albus* nodules in Tunisia as belonging to the genera *Agrobacterium*, *Rhizobium*, and *Neorhizobium*. Although the *nodA* and *nodC* genes could not be amplified, infectivity tests showed nodulation and substantial increases in the dry weight of shoots [43].

In summary, the phylogenetic analysis based on 16S rRNA gene sequences (Figure 1) included the available sequences of *Lupinus* microsymbionts and grouped them into five different clades. Within the genus *Bradyrhizobium*, eight subgroups can be differentiated, related to *B. cytisi, B. hipponenese, B. rifense, B. iriomotense/stylosanthis, B. diazoefficiens, B. japonicum, B. canariense /B. lupini*, and the extra-slow-growing *B. retamae and B. valentinum,* respectively. The strains in the *Microvirga* cluster showed good identity with different *Microvirga* species. In the *Phyllobacterium* cluster, *Phyllobacterium* sp. LmiT21 and *P. trifolii* grouped with *P. endophyticum. Ochrobactrum lupini* was close to *O. cytisi,* and *Devosia* sp. LanTb5 grouped with other *Devosia* spp.

Besides nodulating bacteria, legume nodules often harbor other non-nodulating endophytic bacteria whose role in symbiosis is not clearly defined [3]. Some nodule endophytes have been described as plant growth-promoting rhizobacteria (PGPR). To cite a few examples of lupin nodule endophytes, a *Paenibacillus glycanilyticus* strain isolated from *L. luteus* nodules has been reported to modulate the lipidic and phenolic profile in white lupin, which points to PGPR inoculation as a possible strategy to enhance the nutritional quality of lupin seeds by increasing the quantity and quality of lipids and enhancing their phenolic profile [86]. *Paenibacillus* has also been reported as a nodule endophyte in *L. albus* [87]. Bacteria isolated from *L. luteus* and *L. angustifolius* have been reported to cluster with strains in the genera *Rahnella, Serratia, Raoultella, and Stenotrophomonas* [88]. Most of the endophytes isolated from *L. mutabilis* nodules belong to the family *Bacillaceae* [89]. A potentially new species of *Micromonospora* has been isolated from *L. angustifolius* nodules, and a *nifH*-like gene which was amplified from *Micromonospora* that presented a 99% identity with the *nifH* gene of *Frankia alni*, an actinobacterium that induces nitrogen-fixing nodules in plants within the genus *Alnus* [90]. *Micromonospora* bacteria have also been identified in nodules formed by *A. glutinosa*, *A. viridis,* and other actinorhizal plants [91], where they may have acquired the *nifH* gene from *Frankia*. Horizontal transfer of symbiotic genes from symbiotic to non-symbiotic nodule bacteria has also been reported in different legumes [92,93]. The physical proximity of nodule endophytes with symbiotic bacteria appears to make them likely recipients of symbiotic genes by horizontal transfer.

## 4. Horizontal Transfer of *nod* and *nif* Genes: On How to Become a Lupin Microsymbiont

Depending on the rhizobial genus, symbiotic genes are located in the chromosome (*Bradyrhizobium*), in “symbiotic islands” within the chromosome (*Mesorhizobium*), or in plasmids (*Rhizobium, Ensifer*) [94]. These genes are easily interchangeable among bacteria by lateral or horizontal gene transfer [95], and they can be used to define symbiovars within one or several rhizobial species according to the legume host specificity [37]. Nodulation (*nod*) genes are involved in nodule organogenesis in plant roots and stems. They are exclusive of symbiotic bacteria, and the nodulation (*nodA*, *nodB*, *nodC,* and *nodD*) gene-based phylogenies are normally similar to each other, but substantially differ from the 16S rRNA gene phylogeny [96].

Previous works have shown that the *nod* gene phylogenies somewhat correlate with the host legume [9,96,97]. The *nodC* gene is the most common nodulation gene marker, as this *nod* gene is essential for nodulation in all rhizobial species. On the contrary, *nif* genes are present in many bacteria besides rhizobia, and are not unique to bacteria that are able to fix nitrogen. Nevertheless, it is not clear whether *nif* genes are an evolutionary part of the rhizobial genome or belong to the common bacterial gene pool [98]. It has been reported that the phylogeny of the *nifH* gene is similar to that of the 16S rRNA gene, which suggests that both genes are likely to share the same evolutionary history [98,99]. However, when horizontal transfer of *nif* genes has taken place, a phylogenetic discordance appears [100]. As indicated above, there are various bacteria previously considered as non-symbiotic that have been described as effective lupin microsymbionts, which appear to have acquired their *nod* and *nif* genes most likely through horizontal gene transfer [9,12,84].

### 4.1. nodC, an Essential Gene for Nodulation and Host Specificity

**Figure 2:**
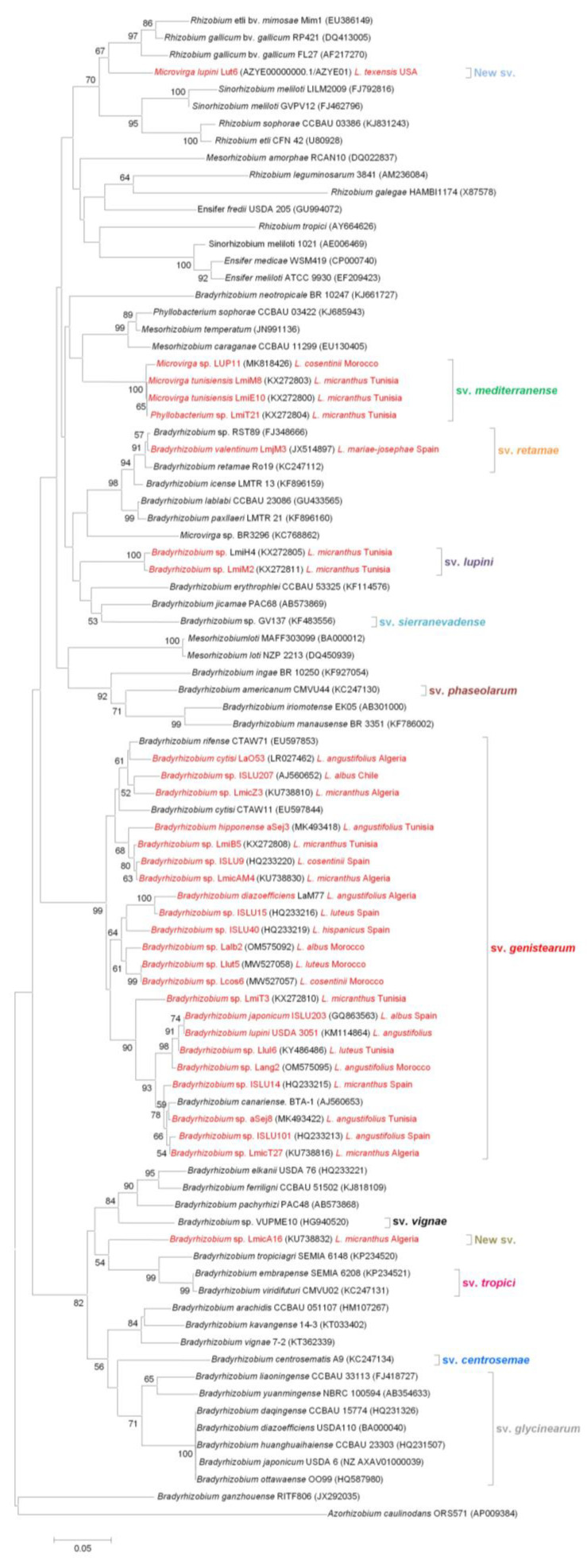
Neighbor-joining phylogenetic analysis of *nodC* gene sequences of different species of *Lupinus* spp. microsymbionts (highlighted in red) and their closest relatives. Bootstrap probability values greater than 50% are indicated.

The symbiotic *nodC* gene encodes for N-acetylglucosaminyltransferase, a protein required for Nod factor assembly. Nod factors are molecules that determine host specificity and are indispensable for nodulation of compatible host legumes [23,37,101]. The *nodC* gene has been used to define the host range and symbiovars within the several rhizobial genera [3,9,37]. *Nod* genes are often considered not useful to classify species of rhizobia due to the possibility of natural horizontal gene transfer among bacterial strains in the soil. However, they prove useful for defining symbiovars. The *nodC* gene-based phylogeny of lupin-nodulating symbiotic rhizobia is presented in Figure 2. This analysis of the available *nodC* sequences from *Lupinus* spp. isolates allows for the identification of six different symbiovars: four of them include *Bradyrhizobium* isolates, one includes the *Phyllobacterium* and *Microvirga* strains, and one includes *M. lupini*. The majority of lupin-nodulating bacteria belong to the sv. *genistearum,* first described by Vinuesa et al. [73], which also includes some strains that nodulate other legume genera in the tribe Genisteae. The sv. *genistearum* is phylogenetically divergent from the rest of *Bradyrhizobium* symbiovars, which are integrated by *Bradyrhizobium* species that nodulate other legumes, namely sv. *glycinearum* [73], sv. *retamae* [102], sv. *vignae* [103], sv. *sierranevadense* [104], sv. *centrosemae* [105], sv. *tropici* [105], and sv. *phaseolarum* [105]. *L. mariae-josephi*-nodulating rhizobia, isolated in Spain, represent a new symbiotic lineage affiliated to sv. *retamae,* which also includes *B. retamae* [39]. Interestingly, all other rhizobial strains isolated from *Lupinus* spp. in the Iberian Peninsula belong to the genera *B. japonicum* and *B. canariense,* within sv. *genistearum*. Based on their symbiotic characteristics and *nodC* sequences, three strains isolated from *L. micranthus* nodules in Tunisia allowed for two new symbiovars to be defined: sv. *lupini*, which comprises two *Bradyrhizobium* isolates, and sv. *mediterranense,* which includes *Microvirga* and *Phyllobacterium* species [9]. Finally, *Bradyrhizobium* sp. LmicA16 and *M. lupini* Lut6 seem to represent to two new symbiovars, which appear distant from all other defined symbiovars. *M. lupini* is close to *Rhizobium* and *Sinorhizobium* (syn. *Ensifer*) strains, suggesting that its *nodC* was obtained from these rhizobial species through horizontal transfer. This is in agreement with a previous study in which a *nodA* gene sequence phylogenetic analysis placed *M. lupini* in the same clade with *Rhizobium*, *Mesorhizobium,* and *Sinorhizobium* species [84]. The *nodC* gene sequences of *Ochrobactrum lupini and Phyllobacterium lupini* are not available. However, their *nodD* gene sequences show a high similarity to the *nodD* gene of *Rhizobium* species [27,41], suggesting that their nodulation genes could have been acquired by horizontal gene transfer from this rhizobial genus. 

When we compare the phylogenetic trees of the *nodC* gene (Figure 2) and the 16S rRNA gene (Figure 1), we can appreciate some consistencies and inconsistencies. Most lupin-nodulating *Bradyrhizobium* species group together within sv. *genistearum*, which suggests that they share a common evolutionary history or that a horizontal transfer of symbiotic genes between different *Bradyrhizobium* species associated with lupin and other *Genisteae* legumes has occurred. However, *B. valentinum*, as well as *Bradyrhizobium* sp. strains LmiH4, LmiT2, and LmicA16, which appear relatively close in the 16S rRNA tree, belong to three quite distant symbiovars according to their *nodC* sequences, all of them still close to bradyrhizobia that nodulate other legumes. Unlike the species in the genus *Bradyrhizobium*, non-rhizobial species isolated from lupin nodules appear to have obtained their symbiotic genes from other rhizobial genera via horizontal transfer [29]. Some *Phyllobacterium* and *Microvirga* strains share a common symbiovar in the proximity of *Mesorhizobium* species, suggesting that they acquired their symbiotic genes from rhizobia within the genus *Mesorhizobium*. These strains were isolated from *L. micranthus* in Tunisia and Morocco, so they share host plant and have close geographical origins. However, *Microvirga lupini*, isolated from *L. texensis* nodules in Texas, represents a completely different symbiovar. According to the *nodC* tree, it could have acquired this gene from phylogenetically close *Rhizobium* or *Ensifer* species.

### 4.2. nifH, a Gene Required for Effective Nitrogen Fixation

**Figure 3:**
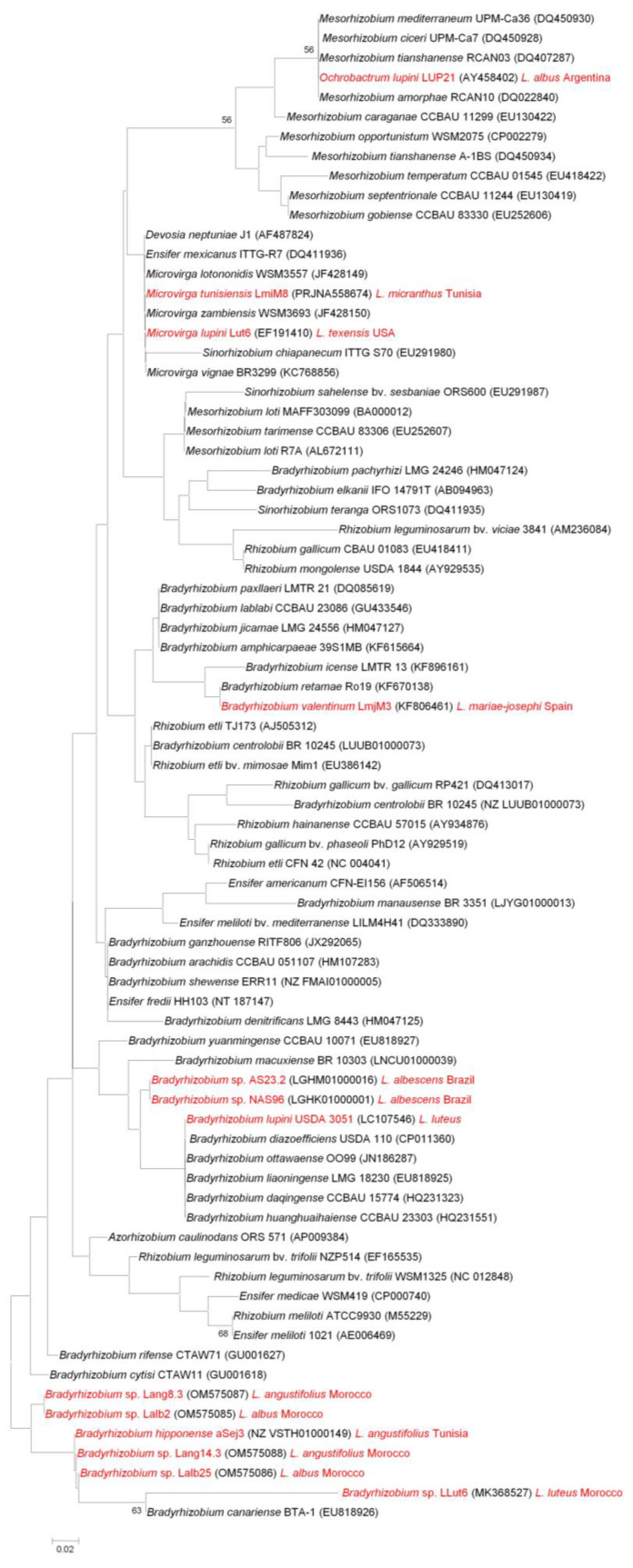
Neighbor-joining phylogenetic analysis of *nifH* gene sequences of different species of *Lupinus* spp. microsymbionts (highlighted in red) and their closest relatives. Values of bootstrap probability greater than 50% are indicated.

The *nif* genes, which codify for different nitrogenase enzyme complex subunits and several regulatory proteins, are required for nitrogen fixation [22]. They are present in rhizobia and in other bacteria capable of nitrogen fixation, including other symbiotic bacteria, such as *Frankia*; endophytes, such as *Azospirillum*; heterotrophic free-living bacteria, such as *Azotobacter*; and cyanobacteria, such as *Anabaena,* but they are also found in bacteria that are not able to fix nitrogen. The *nifH* symbiotic gene is one of the essential genes required to synthesize the enzyme nitrogenase, and it is considered as a highly conserved gene [106]. The *nifH* gene-based phylogenetic tree shown in Figure 3 includes the available *nifH* sequences of lupin-nodulating rhizobia and the *nifH* sequences with the highest similarity. Most strains associated with the genus *Bradyrhizobium* were isolated from lupin species growing in the Old World. American lupin microsymbionts isolated from *L. albescens* are somewhat distant from the Old World group. This is in agreement with a recent report in which a phylogenetic analysis of the *nifD* gene sequences of bradyrhizobia present in Brazilian soils was performed [107]. This analysis clustered the indigenous and the Old World-imported lupin-nodulating *Bradyrhizobium* strains in different clades. The fact that Old World genistoids are predominantly nodulated by Old World bradyrhizobia in American soils suggests that high symbiotic specificity is the reason why microsymbionts have been codispersed and adapted to the soils where their legume hosts have been introduced [107]. *B. lupini* appears closer to the American strains than to the Old World group, while *B. valentinum* is distant from the rest of lupin microsymbionts, and clusters together with *B. retamae* in concordance with the *nodC* gene phylogenetic analysis (Figure 2). 

Regarding lupin-nodulating bacteria not belonging to the genus *Bradyrhizobium*, *Microvirga tunisiensis* groups together with *M. lupini* despite the fact that their respective *nodC* gene sequences seem to have different origins (Figure 2). They appear close to *M. lotononidis* and *M. zambiensis,* isolated from *Listia angolensis* nodules, a species within the only other genus besides *Lupinus* that forms lupinoid nodules. This suggests that *Microvirga* species share their own *nifH* gene. A phylogenetic analysis based on the *nifD* and *nifH* gene sequences of *M. lupini*, *M. lotononidis,* and *M. zambiensis* showed that these species are closely related to *Rhizobium etli* [84]. The *nifH* sequence of *Ochrobactrum lupini* isolated from *L. honoratus* nodules [27] shows high similarity to those of several *Mesorhizobium* species, which suggests that *O. lupini* obtained its symbiotic genes by horizontal transfer from *Mesorhizobium*.

## 5. Conclusions

Most *Lupinus* spp. microsymbionts isolated all over the world belong to the *Bradyrhizobium* lineage. While all lupin-nodulating rhizobia were initially referred to as *Bradyrhizobium* sp. (*Lupinus*) and sometimes as *B. lupini*, the advancement of molecular biology, bioinformatics tools, and gene sequence-based taxonomy has allowed for the identification of numerous species under the *Bradyrhizobium* sp. (*Lupinus*) umbrella, as shown in the phylogenetic analysis based on 16S rRNA gene sequences (Figure 1). This analysis groups lupin microsymbionts into five different groups. The largest one corresponds to the genus *Bradyrhizobium*, and comprises eight subgroups. Four additional clades include the fast-growing bacteria *Microvirga, Phyllobacterium, Ochrobactrum,* and *Devosia*. Bacteria associated with the genera *Agrobacterium*, *Rhizobium, and Neorhizobium* have also been described as lupin microsymbionts according to a concatenated phylogenetic analysis which used the sequences of housekeeping genes. However, the 16S RNA gene sequences of these strains are not yet available.

It is a known fact that, in general, the phylogeny of symbiotic genes is not in congruence with that of core genes (16S rRNA and housekeeping genes) [21]. Symbiotic genes’ phylogenies can be used to characterize isolates at the symbiovar level [37]. Divergences between phylogenies indicate that horizontal gene transfer of symbiotic genes might have occurred between species and/or genera. Full genome sequence analysis of symbiotic bacteria will likely increase our understanding of the legume–rhizobia interactions, and, most probably, will lead to a modification of the taxonomic criteria currently accepted for the definition of rhizobial species and genera. In fact, the taxonomy of bacteria that induce nodules in *Lupinus* spp. has been rearranged in recent years with the definition of new species and genera, the reclassification of some species in new genera, and the definition of new symbiovars. Moreover, a significant increase in the number of genera and species of lupin microsymbionts is expected to occur in the future, considering that most studies to date are mostly limited to Old World lupin species, while between 200 and 500 New World *Lupinus* species remain to be characterized.

The phylogenetic analysis of *nodC* sequences (Figure 2) suggests the existence of six different symbiovars: four of them include *Bradyrhizobium* isolates, one includes *Phyllobacterium* and *Microvirga* strains, and one includes *Microvirga lupini*. The majority of *Lupinus* spp. symbiotic bacteria in the genus *Bradyrhizobium* which are related to *B. lupini* and to the phylogenetically close *B. cytisi/B. rifense* lineages belong to the symbiovar *genistearum*. However, some lupin-nodulating *Bradyrhizobium* species appear to constitute new distant symbiotic lineages, namely sv. *retamae* and sv. *lupini,* and, possibly, a new symbiovar. Symbiovar *mediterranense* comprises *Microvirga* and *Phyllobacterium* strains, and *M. lupini* appears to represent another new symbiovar. Despite their taxonomic distance, it seems that the *nodC* symbiotic genes of all bradyrhizobia that nodulate lupin are more closely related to other *Bradyrhizobium* species than to other rhizobial genera. Contrary to lupin-nodulating bradyrhizobia, it appears that other lupin microsymbiont species have acquired their *nod* genes from several rhizobial genera other than *Bradyrhizobium*. The *nifH* gene-based phylogenetic tree (Figure 3) suggests that within the genus *Bradyrhizobium*, this gene seems to somehow be associated with the geographical origin of the strains. In the *nifH* tree, all *Microvirga* species cluster together, suggesting that, contrary to the foreign origin of their *nodC* gene, they share a common *nifH* gene which might not have been obtained from other rhizobia through horizontal transfer. The *nifH* gene of *Ochrobactrum lupini* is probably the result of a horizontal transfer event from *Mesorhizobium* species.

Several studies have reported the isolation of *Paenibacillus, Micromonospora, Rahnella, Serratia, Raoultella,* and *Stenotrophomonas* strains from lupin nodules; however, these bacterial isolates have not been confirmed to form effective nitrogen-fixing nodules. Most of these nodule endophytes have been reported to have PGPR traits. The horizontal gene transfer from symbiotic bacteria to endophytes represents a one-step evolution that might lead to the emergence of new symbiotic bacteria; therefore, it is ecologically and evolutionarily important [108]. Despite the physical proximity of endophytes and symbiotic bacteria in lupin nodules, it seems unlikely that they gain nodulating and nitrogen-fixing capacities from bradyrhizobia, as no horizontal transfer of symbiotic genes from *Bradyrhizobium* to other lupin-nodulating bacteria has been described to date. However, the transfer of *nifH* from soybean-nodulating *B. japonicum* to endophytic *Bacillus* has been reported [109], and still, the possibility exists that lupin nodule endophytes acquire symbiotic genes when sharing nodule occupancy with lupin microsymbionts other than *Bradyrhizobium*.

Overall, the data that are available so far and included in the present review suggest that horizontal transfer of specific symbiotic genes from different rhizobial genera represents a key mechanism, allowing lupin to form symbioses with selected bacteria initially considered as non-symbiotic or unable to nodulate lupin. These new lupin microsymbionts are probably better adapted to particular soil and climate conditions. Thus, the dissemination of indiscriminate symbiotic genes appears to be part of a bacterial cooperation strategy which benefits a biodiverse population rather than a particular species, and allows for the development of strain-specific legume–rhizobia symbioses in particular habitats, which would not be viable otherwise. A recent review [29] reported on the occurrence and importance of horizontal transfer of symbiotic genes within and between rhizobial genera, addressing the diversity of microsymbionts that are able to nodulate different legume genera. The authors did not report, however, on lupin and its nodulating bacteria. Lupin is a singular legume crop in many ways, and is, foremost, a promising yet underexploited plant protein source. It also presents great potential as a medicinal plant, owing to its seed particular protein, lipidic, and phenolic profiles. Its application in the remediation of poor and degraded soils and heavy metal-contaminated soils has also been proposed as very promising. Well-adapted microsymbionts have been shown to increase lupin tolerance to heavy metals, and certain inoculants have been reported to improve the nutritional and antioxidant characteristics of lupin seeds. In the present review, we summarized the current knowledge on the diversity of microsymbionts that nodulate *Lupinus* spp., and the important role that horizontal transfer seems to play. Our conclusions are in agreement with those of Andrews et al. [29]. Deeper insight into the nature of lupin microsymbionts, including comprehensive whole genome analyses, will undoubtedly provide new knowledge that might prove useful for the purpose of increasing lupin’s stress tolerance, adaptability to marginal soils and climates, grain quality, and crop yields.
